# Proteomic differences between native and tissue‐engineered tendon and ligament

**DOI:** 10.1002/pmic.201500459

**Published:** 2016-05-11

**Authors:** Yalda A. Kharaz, Simon R. Tew, Mandy Peffers, Elizabeth G. Canty‐Laird, Eithne Comerford

**Affiliations:** ^1^ Department of Musculoskeletal Biology, Institute of Ageing and Chronic DiseaseUniversity of LiverpoolLeahurst CampusNestonUK; ^2^ The MRC‐Arthritis Research UK Centre for Integrated Research into Musculoskeletal Ageing (CIMA)LiverpoolUK

**Keywords:** 3D tissue engineered ligament construct, 3D tissue engineered tendon constructs, Ligament, Proteomics, Tendon, Technology

## Abstract

Tendons and ligaments (T/Ls) play key roles in the musculoskeletal system, but they are susceptible to traumatic or age‐related rupture, leading to severe morbidity as well as increased susceptibility to degenerative joint diseases such as osteoarthritis. Tissue engineering represents an attractive therapeutic approach to treating T/L injury but it is hampered by our poor understanding of the defining characteristics of the two tissues. The present study aimed to determine differences in the proteomic profile between native T/Ls and tissue engineered (TE) T/L constructs. The canine long digital extensor tendon and anterior cruciate ligament were analyzed along with 3D TE fibrin‐based constructs created from their cells. Native tendon and ligament differed in their content of key structural proteins, with the ligament being more abundant in fibrocartilaginous proteins. 3D T/L TE constructs contained less extracellular matrix (ECM) proteins and had a greater proportion of cellular‐associated proteins than native tissue, corresponding to their low collagen and high DNA content. Constructs were able to recapitulate native T/L tissue characteristics particularly with regard to ECM proteins. However, 3D T/L TE constructs had similar ECM and cellular protein compositions indicating that cell source may not be an important factor for T/L tissue engineering.

**Abbreviations**
3D: three dimensional
ACL: anterior cruciate ligament
ALC: average local confidence score
ECM: extracellular matrix
LDET: long digital extensor tendon
LF: label free
sGAG: sulphated glycosaminoglycan
TE: tissue engineered
TEM: transmission electron microscopy
T/L: tendon and ligament

## 1 Introduction

Tendons and ligaments (T/Ls) are dense connective tissues that play key roles in musculoskeletal system. Both tissues have specialized functions required for efficient locomotion 1. T/L injuries are increasingly common in humans, in comparative species such as the dog and horse 2, 3, 4, 5 and are caused as result of degeneration or trauma/acute tears. There are currently more than 30 million tendon injures per year worldwide 6, with 30–50% of these being sports related 7. With regards to tendon injuries, rotator cuff tears in humans 8 and superficial digital flexor tendinopathy in the horse 9 are the most common. The ethiopathogenesis of this tendinopathy is thought to be caused by repetitive micro trauma resulting in degenerative changes subsequently leading to injury 1. The anterior cruciate ligament (ACL) is one of the most frequently injured ligaments in humans with 30% caused by trauma and 70% due to degenerative and noncontact injuries 10. Both ACL and medial collateral ligament injuries account for 95% of all multiligament injuries in the knee joint 11, resulting in significant joint instability and morbidity 12. Ligament injury can also lead to significant functional impairment in athletes resulting in degenerative joint diseases such as osteoarthritis (OA) 13, 14. Severe T/L injuries are presently treated with autografts or allografts, but these are associated with complications such as infection 15, disease transmission and graft rejection 15, 16, chronic pain 17, decreased muscle strength 18, and donor site morbidity 19.

**Significance of the study**

Tissue engineering approaches have the potential to provide materials for treatment of tendon and ligament injuries. To date, no studies have characterized the proteome of engineered tendon or ligament using the increasingly popular 3D fibrin‐based culture system. In this paper, we report the first proteome profile of 3D tendon and ligament TE constructs and have performed a comprehensive proteomic analysis to reveal differences between constructs and native tissues.

Tissue engineering offers great potential for the treatment of T/L injury by aiming to provide a biological replacement that mimics the structure, function, and longevity of native tissue 20. The tissue engineering approach involves the acquisition and cultivation of an adequate source of cells, addition of growth inducing stimuli and provision of an artificial extracellular matrix (ECM) (scaffold) in which cells can proliferate and differentiate enabling new tissue generation 21. Fibrin is a natural biomaterial that has been used for the creation of engineered T/L constructs using variety of cell sources including rat and chick tendon fibroblasts 22, 23, 24, 25, human bone marrow stem cells 26, human adult ACL 27, and tendon cells 28. To date an understanding of the proteins that comprise these tendon and ligament‐like structures is unknown. The hypotheses of this study were that (i) native tendons and ligaments have different structural protein content and that (ii) three dimensional (3D) tissue engineered (TE) constructs formed from T/L cells possess the proteome characteristics of the original tissues. Therefore, this study aimed to identify the differences between the proteomes of native T/L as well as those from engineered T/L 3D constructs. In this study, a proteomics workflow using a gel‐free separation technique with label‐free (LF) quantification was used to identify differences in protein abundance. A detailed proteomic comparison between native and TE tendons and ligaments has not previously been reported.

## 2 Materials and methods

All chemicals were supplied by Sigma–Aldrich, Dorset, UK unless otherwise stated.

### 2.1 Tissue collection and preparation

ACLs and long digital extensor tendons (LDETs) were harvested immediately after euthanasia from five‐paired cadaveric canine knee joints. The knee joints were from skeletally mature Staffordshire bull terrier dogs (2–5‐year‐old) with a healthy body score (4–5/9). The joints were assessed as disease free by gross inspection. The dogs were euthanased for purposes not related to this study and ethical approval for use of the cadaveric material was granted by Veterinary Research Ethics Committee, School of Veterinary Science (VREC64). Tissues from the right knee joint were used for protein isolation and proteomic analysis of the native tissues. Tissues from the left knee joint were used for cell isolation and creation of engineered tissues, which were subsequently used for protein extraction and proteomic analysis. All samples were snap frozen in liquid nitrogen and stored at −80°C until required.

### 2.2 Tendon and ligament 3D TE construct formation

The 3D TE constructs were created using a 3D fibrin‐based culture system with isolated ACL and LDET cells as described previously 22 with minor modifications as detailed in the Supporting Information Methods.

### 2.3 Protein extraction

Native tendon, ligament, and harvested T/L constructs samples were freeze dried overnight and the dry weight then measured. Approximately, 3 mg of each lyophilized sample was disrupted using a microdismembrator (B.Braun Biotech. International, Germany). Each sample was digested with 1 U/mL chondroitinase ABC in 100 mM tris acetate pH 8, containing mini protease inhibitor cocktail with EDTA (Roche, UK) for 6 h at 37°C with end over end mixing. The supernatant was removed following centrifugation at 15 000 rpm at 4°C for 15 min. Tissue was extracted in 500 μL 4 M guanidine hydrochloride (GnHCl), 65mM dithiothreitol, 50 mM sodium acetate, pH 5.8 for 48 h at 4°C with shaking. The samples were then centrifuged at 15 000 rpm at 4°C for 15 min and the soluble fraction was removed. The protein concentration of each soluble fraction was estimated using a Pierce^TM^ 660 nm protein assay (Thermo Scientific, Hertfordshire, UK) and aliquots analyzed by 1D SDS‐PAGE gel electrophoresis to grossly assess the protein expression profile between samples.

### 2.4. In‐solution trypsin digestion

Prior to trypsin digestion the GnHCL soluble fraction was diluted eightfold with 100 mM ammonium bicarbonate and further normalized to the sample that had the lowest protein concentration. In‐solution tryptic digestion was carried out as described previously 29.

### 2.5 LC–MC/MC

LC–MS/MS was performed using an Ultimate 3000 nano system (Dionex/Thermo Fisher Scientific) coupled online to a Q‐Exactive Quadrupole‐Orbitrap instrument (Thermo Fischer Scientific) using 10 μL aliquots of tryptic peptides equivalent to 93 ng protein per sample. Samples were randomized and run on a 1 h gradient with 30 min blanks in between runs as detailed in the Supporting Information Methods.

### 2.6 Proteomic data analysis

MS data were analyzed for identification of protein composition and LF quantification using PEAKS (Version 6, Bioinformatics Solutions, Waterloo, Canada) and Progenesis^QI^ LC‐MS (Waters, Elstree Hertfordshire, UK) software. MS data are available in PRIDE database (http://www.ebi.ac.uk/pride/) at the European Bioinformatics Institute under accession number PXD003094.

To identify the protein composition in each group (native ligament, native tendon, 3D ligament and 3D tendon) raw MS/MS data were imported into PEAKS and searches then performed against the Ensembl canine taxonomy (http://www.ensembl.org/info/data/ftp/index.html).

Instrument configuration was set as Orbitrap (Orbi‐Orbi) and high energy collisional dissociation fragmentation. The following parameters were used for the PEAKS search; parent mass error tolerance, 10 ppm; fragment mass error tolerance, 0.1 Da; precursor mass search type, monoisotypic; enzyme, trypsin; max missed cleavage, 1; nonspecific cleavage, 1; fixed modification; carbamidomethylation, variable modification; oxidation, methionine, hydroxylation, and variable PTMs per peptide, 3. The results were filtered on the basis of the following parameters; de novo average local confidence score percent threshold, 50; protein –10lg*p* > 20; FDR at peptide spectrum matches, 1%; and unique peptides ≥2. The ensemble protein accessions were input into Ingenuity Pathway Analysis (IPA, Ingenuity Systems, Redwood City, CA, USA) and gene symbol with protein description and protein subcellular locations were then mapped. Proteins were classified into ECM categories according to the Matrisome Project 30, 31. The remaining proteins were categorized according to UNIPROT function description 32. GO and protein network analysis was carried out using the String bioinformatics tool, version 10 33. LF quantitative analysis was performed using Progenesis^QI^ LC‐MS software. Search results in PEAKS were adjusted to 1% FDR, unique peptides ≥2 and average local confidence score >50% and search hits were imported into Progenesis^QI^ 34.

### 2.7 Biochemical analysis

The biochemical composition of native T/L tissues or 3D TE T/L constructs (*n* = 5) was determined by measuring double stranded DNA, collagen, and sulphated glycosaminoglycan (sGAG) content using previously described protocols 35, 36, 37.

### 2.8 Histology and immunohistochemistry

Native T/L samples and TE constructs created from isolated cell samples (*n* = 3) were fixed in 4% paraformaldehyde for 48 h, embedded in paraffin wax, and 4 μm longitudinal sections mounted on polylysine‐coated slides. Sections were stained with H&E and Alcian blue‐periodic acid Schiff stain (AB‐PAS) (TCS, Biosciences Ltd., Buckingham, UK) 38.

Immunohistological staining was performed on native T/L tissue for asporin, aggrecan, versican, and collagen type III on deparaffinized sections. The immunohistochemistry procedure and antibodies details are provided in the Supporting Information Methods.

### 2.9 Transmission electron microscopy

Transmission electron microscopy (TEM) of T/L 3D TE constructs (*n* = 3) was performed following fixation in 2.5% glutaraldehyde in 0.1 M sodium cacodylate buffer (Agar Scientific, Essex, UK) for 8 h, followed by a second fixation and contrast stain with 0.1% osmium tetroxide for 90 min. Samples were stained with 8% uranyl acetate in 0.69% maleic acid for 90 min, dehydrated in ascending ethanol concentrations, and embedded in epoxy resin (all from TAAB Laboratories Equipment Ltd., Berks, UK). Ultrathin cross‐sections (60–90 nm) were cut with a Reichert‐ Jung Ultracut ultramicrotome (Leica Microsystems Ltd., Milton Keynes, UK) using a diamond knife. Sections were then mounted on 200 mesh copper grids and stained with “Reynold's Lead citrate” stain (VWR, Leicestershire, UK) for 4 min. Images were obtained using a Philips EM208S Transmission Electron Microscope at 80 KV.

### 2.10 Statistical analysis

Statistical analysis for proteomic LF datasets was performed by Progenesis^QI^ on all detected features using transformed normalized abundances for one‐way ANOVA. Identification of proteins with two or more peptides, greater than twofold abundance and with a *q* value (*p*‐value adjusted to FDR) <0.05 were considered significant. Quantitative analysis was initially performed by comparing the four groups of tissue samples together. After that pair‐wise comparisons were performed between native ligament and tendon, native ligament and 3D TE ligament construct, native tendon and 3D TE tendon construct, and 3D TE ligament and 3D TE tendon constructs. Datasets for biochemical analysis were first assessed for normality using a Kolmogorov‐Smirnov test (Graphpad Software, Version 6, La Jolla, CA, USA). All data sets were normal distributed and were analysed using one‐way ANOVA with a Bonferroni post‐hoc test using Graphpad Prism. The significance level was set at *p* < 0.05.

## 3 Results

### 3.1 Engineered 3D tendon and ligament constructs display a loose architecture with a high degree of cellularity

**Figure 1:**
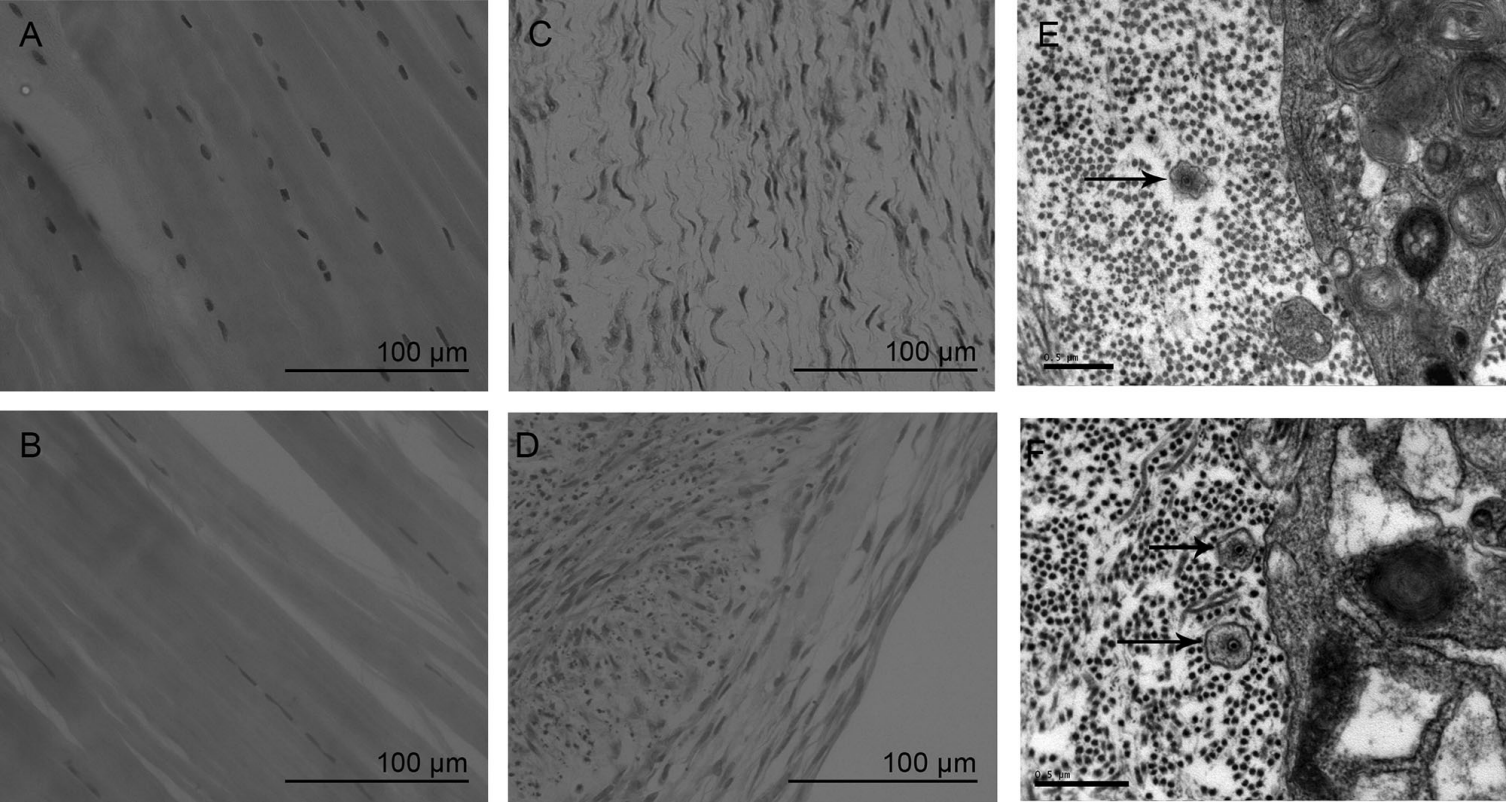
Ultrastructural images of native T/L and 3D TE constructs. H&E staining of native ligament (A), native tendon (B), 3D ligament constructs (C), and 3D tendon construct (D) (Bar 100 μm). Transmission electron microscopy of 3D TE tendon (E) and ligament (F) constructs indicate the presence of aligned extracellular collagen fibrils and fibripositors (black arrows) demonstrating that constructs have formed correctly.

Histological observation of native tendon demonstrated a dense, parallel aligned architecture, and long elongated cellular morphology, however native ligament had less compact collagen fiber alignment and a more rounded cell morphology (Fig. 1A and B). H&E staining of 3D constructs indicated that both tendon and ligament constructs had a loose architecture and a high degree of cellularity with a fibroblastic cellular morphology (Fig. 1C and D). The presence of collagen fibrils was confirmed using TEM, where close‐packed narrow diameter collagen fibrils were visible in the extracellular space (Fig. 1E and F). Collagen fibrils were also found to be located in collagen fibripositors (Fig. 1E and F), which are actin‐rich plasma membrane protrusions that mediate collagen fibril organization in embryonic tendon 39.

### 3.2 Matrisomal proteins and GO terms associated with ECM organization were more strongly represented in native tissue than in engineered 3D constructs

An average protein content of (μg/mg dry weight) of 181 was measured for native T/L and 284 for 3D T/L constructs. A total of 3569, 3743, 4481, and 5790 peptides assigned to 167, 215, 442, and 561 proteins each were identified in native ligament, native tendon, 3D ligament, and 3D tendon, respectively. Between both native tissues and 3D tissues 93 proteins were common, which included several ECM proteins such as collagen type I,III, V,VI, decorin, biglycan, lumican, tenascin C, fibrillin 1, fibulin 1, thrombospondin 1, and cellular proteins such as vimentin, ATP synthase, and actin (Supporting Information Table 1).

**Figure 2:**
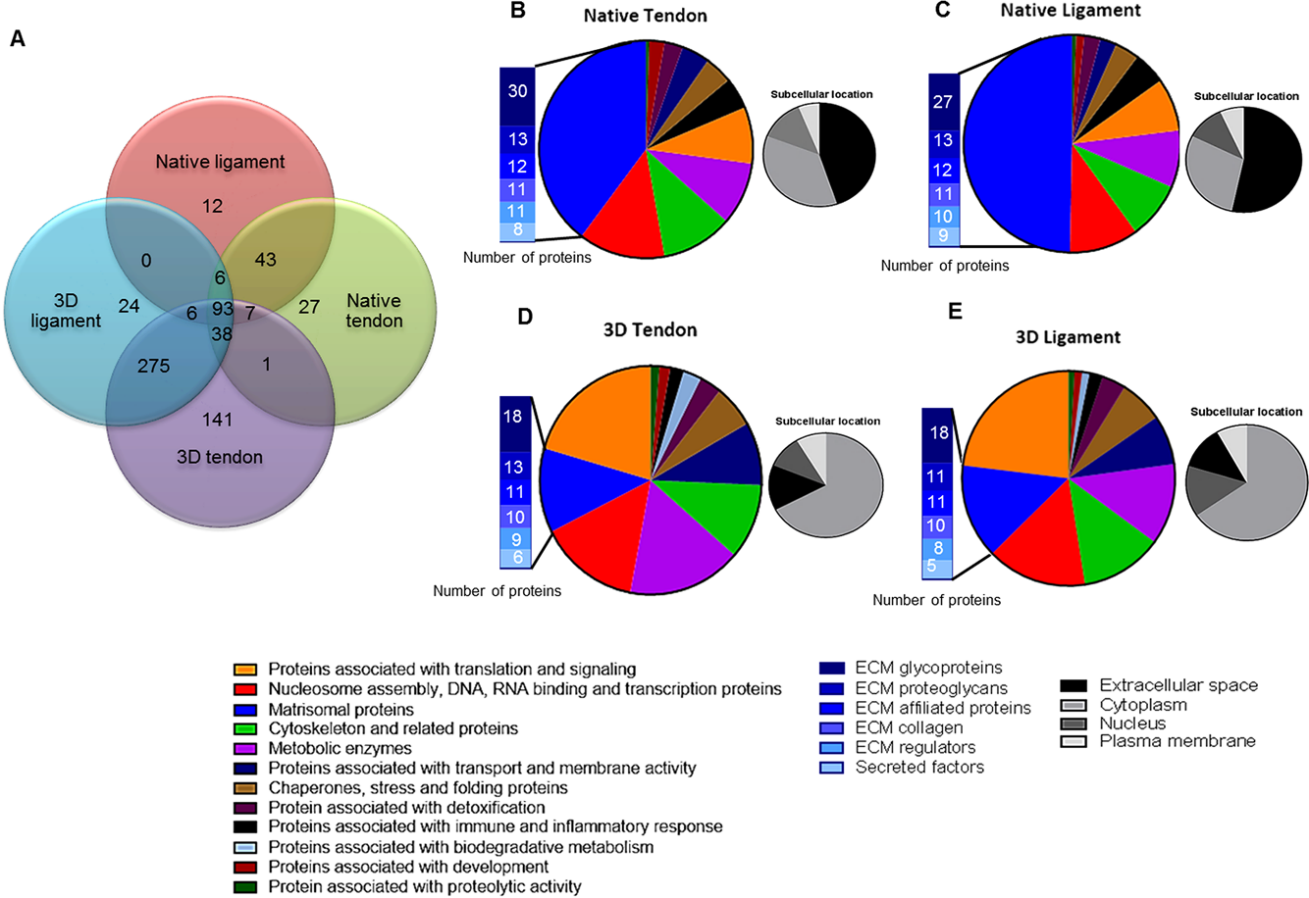
Protein composition of native T/Ls and 3D TE construct identified with PEAKS. The total number of proteins identified following MS in each tissue type as well as common proteins between the tissue types is presented (A). The proteomes of native tendon (B), ligament (C), 3D tendon (D), and 3D ligament (E) constructs were subdivided based on Uniprot function and matrisomal classification (Matrisome Project). The associated subcellular locations of the proteins are also shown (B–E).

The native T/L proteome contained 40 and 50% matrisomal proteins respectively, with 45 and 53% of proteins locations annotated to extracellular space, (Fig. 2B and C). The remainder of the native T/L proteome was associated with cytoplasmic, nucleus, and plasma membrane locations. In both 3D TE tendon and ligament constructs 66.3% of proteins were associated with cytoplasmic location whereas 22% of proteins were associated with translation and signaling and 13.1% were matrisomal proteins (Fig. 2D and E).

STRING protein–protein interaction network analysis and GO in both native T/L tissues resulted in connected clusters around ECM proteins and matrisomal associated proteins. The principal GO processes for both tendon and ligament tissues were identified as ECM organization (FDR adjusted *p*‐values 1.53 × 10^−26^ and 3.75 × 10^−27^, respectively), wound healing (FDR adjusted *p*‐value 1.94 × 10^−14^ and 1.85 × 10^−14^) and collagen fibril organization (FDR adjusted *p*‐values 2.01 × 10^−21^ and1.19 × 10^−13^) (Supporting Information Fig. 1A and B). Principle ontology for 3D ligament and 3D TE tendon constructs involved translational elongation (FDR adjusted *p*‐values 3.71 × 10^−65^ and 2.3 × 10^−63^) and protein targeting to ER (FDR adjusted *p*‐values 9.98 × 10^−64^, and 1.75 × 10^−65^). The strongest predicted protein–protein interaction was between the ribosomal proteins (Supporting Information Fig. 1C and D).

### 3.3 Quantitative differences in protein composition were observed between native tendon and ligament but not between constructs formed from either tendon or ligament cells

**Figure 3:**
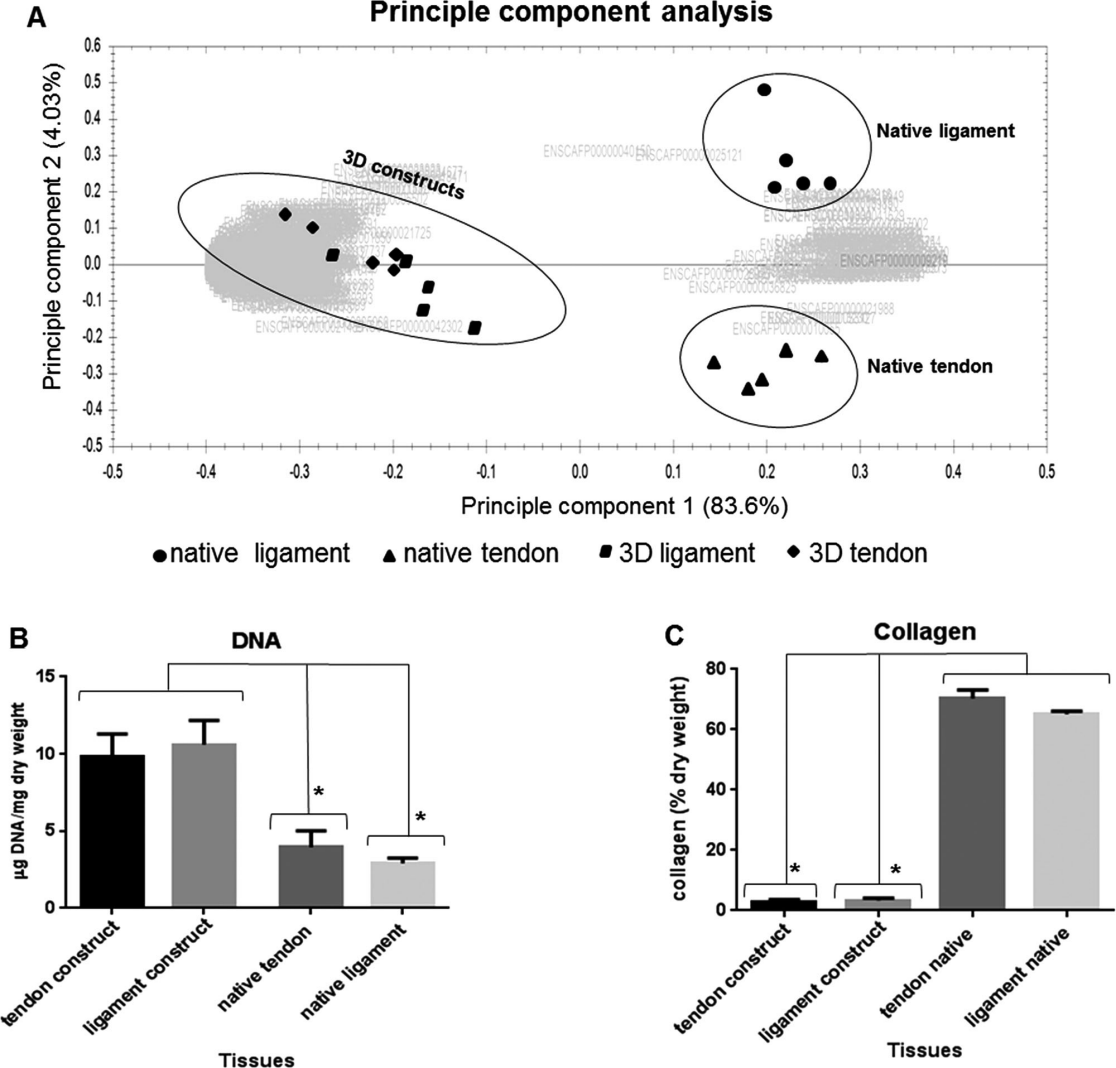
PCA and biochemical composition comparison between native tissues and 3D TE constructs. PCA (A) between native tendon, ligament and 3D TE constructs, and a comparison of DNA (B) and collagen content (C) (% of dry weight) between native tissues and 3D constructs is shown. Values are mean and error bars represent SEM. *p < 0.05.

Quantitative LF analysis demonstrated a set of 387 proteins within the four tissue types with a fold change ≥2 and unique peptides >2. PCA revealed that native ligament and tendon samples were distinctly grouped, whereas 3D tendon and tendon did not cluster into discrete groups (Fig. 3A).

**Figure 4:**
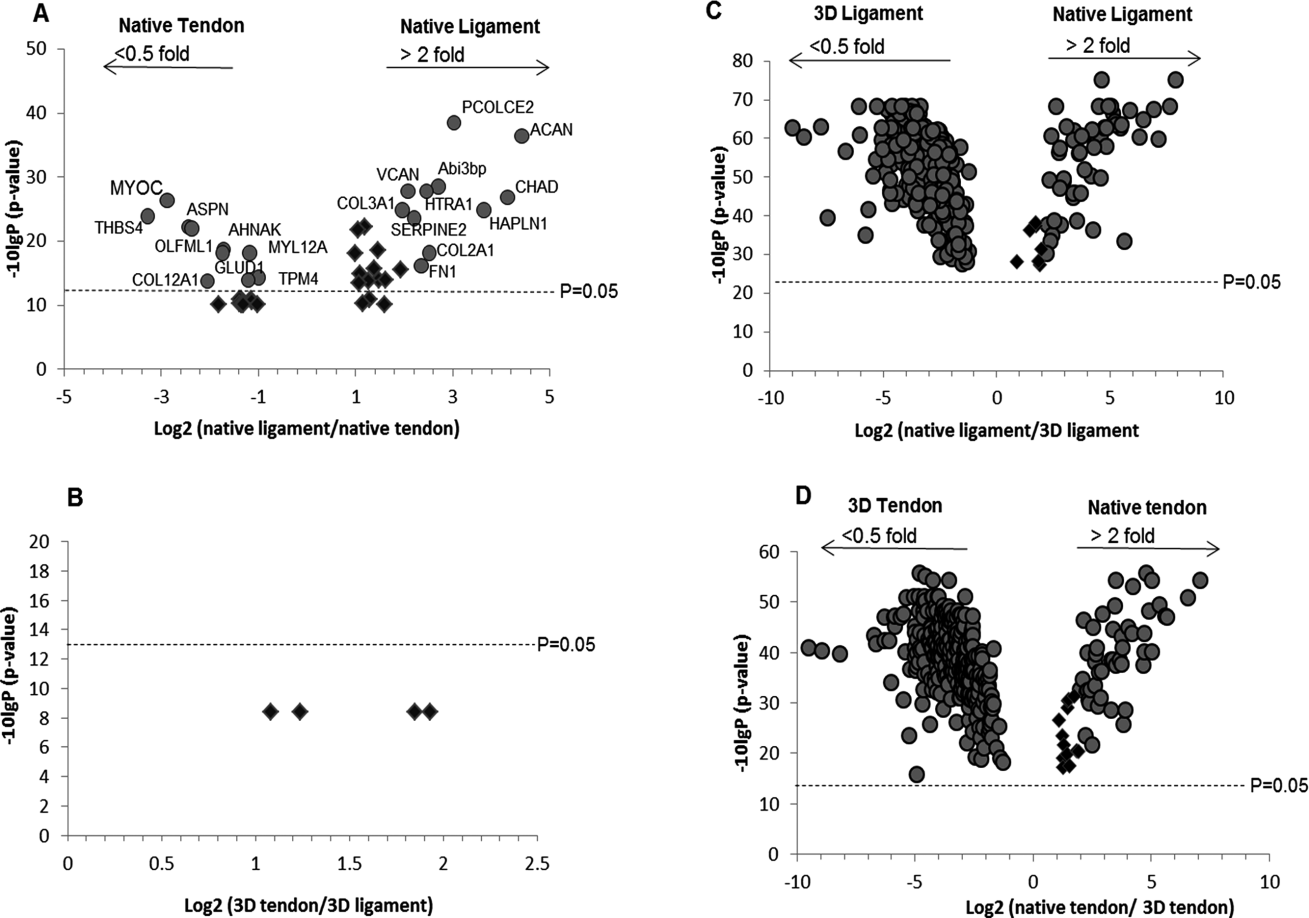
LF proteomic analysis of group comparisons between native tissues and 3D TE constructs using Progenesis^QI^ software. Volcano plots demonstrate proteins that were more abundant and significant (*p* < 0.05) between native ligament versus native tendon (A), native ligament versus 3D ligament (C), and native tendon versus 3D tendon (D). No significant proteins were identified between 3D tendon and 3D ligament construct (B) as all protein *p*‐values were greater than 0.05. Abundant proteins in panel C and D are highlighted in Supporting Information Tables 2 and 3.

Pairwise quantitative comparison between native TL demonstrated that native ligament was more abundant in fibrocartilaginous proteins such as collagen type II, alpha 1, agreccan, and chondroadherin, while tendon had more thrombospondin 4, asporin, and collagen type XII (Fig. 4A). No differentially expressed proteins were found between 3D TL constructs (Fig. 4B). Quantitative differences between native tendon and 3D tendon resulted in 321 and 62 proteins being more abundant in 3D tendon and native tendon respectively. When native ligament and 3D ligament were compared 301 proteins were more abundant in 3D ligament and 62 proteins were more abundant in native ligament (Fig. 4C and D).

Observation of abundant proteins in both native tendon and ligament when compared to 3D tendon and ligament engineered constructs demonstrated not only the presence of significantly more ECM proteins and enzyme enhancers but also more blood/plasma proteins in both native tissues. In contrast both 3D TE constructs had significantly more cellular proteins (Supporting Information Tables 2 and 3).

### 3.4 Biochemical analysis confirmed differences in cellular and collagen content between native tissues and engineered

**Figure 5:**
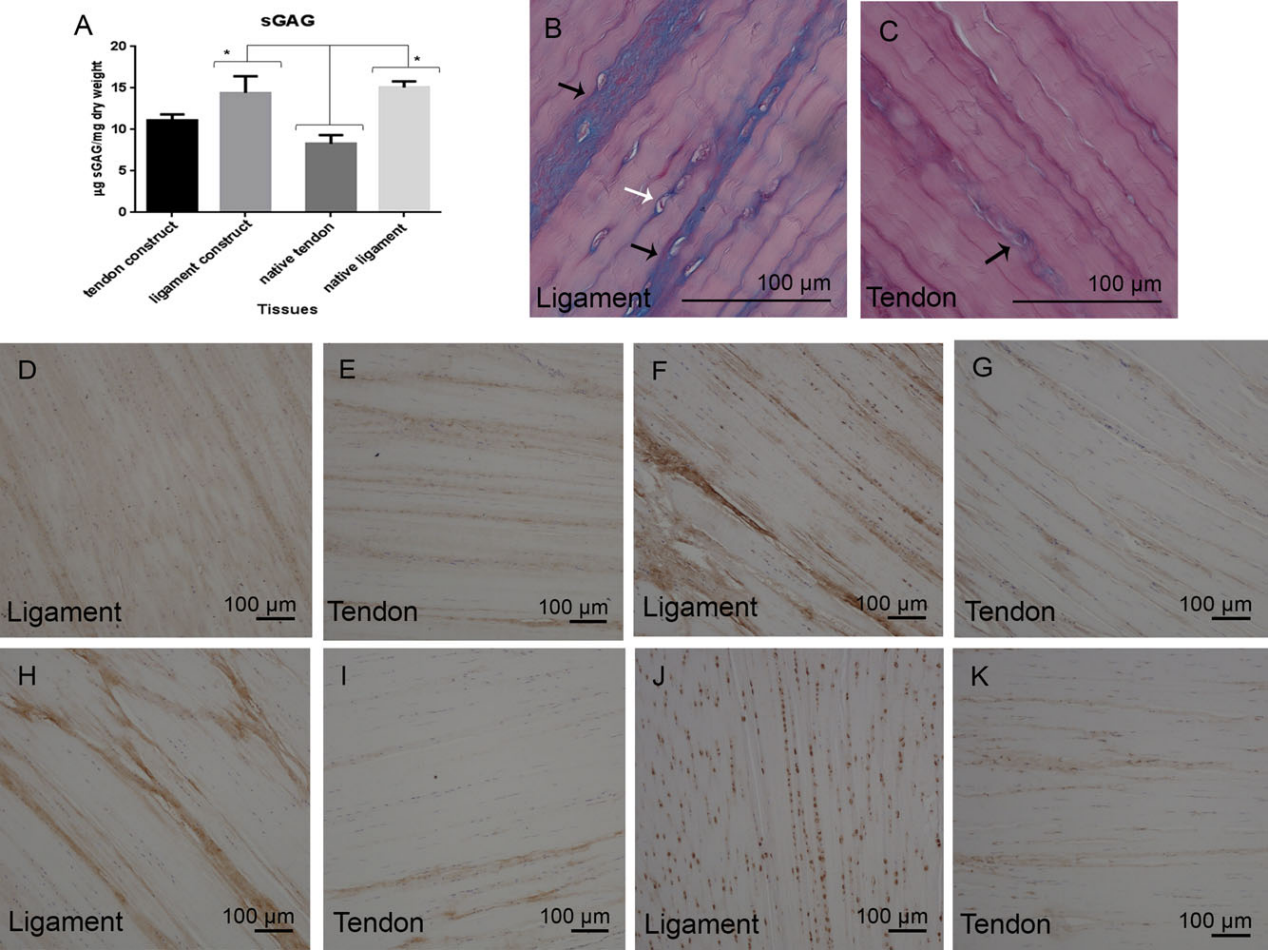
sGAG content in native tendon, native ligament, and 3D TE constructs and validation of proteomic results. sGAG content measurement (A) (μg/mg dry weight) and Alcian blue‐periodic acid Schiff stain histology staining of native ligament (B) and native tendon (C) (Bar 100μm) is demonstrated. Immunohistochemistry staining of native ligament and tendon for collagen type III (D, E), aggrecan (F, G) versican (H, I) and asporin (J, K) (Bar 100 μm). Statistical values represent the mean, error bars represent SEM and *p < 0.05.

Both 3D T/L constructs demonstrated significantly higher DNA content (10.2 ± 1.5 mg/dry weight) indicating a high cellularity of both constructs in comparison to the native tissues (3.43 ± 0.7) (Fig. 3B). In contrast, the collagen content was significantly lower in the constructs (average of 3.2%) compared to the native tissues (average of 67.8%) (Fig. 3C). Native ligament (15.1 ± 0.7) (Fig. 5A) contained significantly more sGAG in comparison to the native tendon (8.3 ± 1.03) where higher GAG staining in native ligament (Fig. 5B and C) was located between collagen fascicles, fibre bundles and surrounding cells. Each 3D TE tendon or ligament construct contained a comparable sGAG content to the native tissue (Fig. 5A). Native ligament (15.1± 0.7) had significantly higher sGAG compared to native tendon. Only 3D ligament constructs (11.1 ± 0.7) had significantly more sGAG than native tendon. No significant differences were found between both 3D TL constructs.

### 3.5 Immunohistochemistry supported differences in the abundance of versican between tendon and ligament and further demonstrated an altered tissue distribution of type III collagen, aggrecan, and asporin

Proteomic comparison identified native ligament to be more abundant in collagen type III, aggrecan, and versican, while tendon contained more asporin. These findings were supported with immunohistochemical staining. In comparison to tendon, collagen type III was differentially distributed in ligament being more widespread in ligament substance while in tendon it was mainly present between collagen fascicles (Fig. 5D and E). A marked presence of aggrecan (Fig. 5F and G) and versican (Fig. 5H and I) was noted in ligament between collagen fascicles in comparison to tendon. Aggrecan was also localized pericellularly in ligament and this could be a key characteristic of ligament cells. Asporin (Fig. 5J and K) was found to be distributed between collagen fascicles and surrounding tenocytes in tendon, while in the ligament asporin was only localized around ligament cells.

## 4 Discussion

In this study, we have performed a comprehensive analysis of the proteomic composition of native T/L tissue and 3D TE fibrin‐based constructs. The results support the hypothesis that there are key structural protein differences between native T/L and that 3D TE constructs share similar characteristics with native tissues particularly with regard to prominent ECM proteins.

The abundance of more fibrocartilaginous proteins such as collagen type II, aggrecan, versican, chondroadherin, and hyaluronic acid link protein in native ligament (ACL) is most likely to be due to the physical adaptation of the tissue against compressive or shear forces generated during twisting of the ACL as the knee joint moves through normal range of motion 40. The formation of fibrocartilage in TLs has been shown to occur in response to compression, primarily in regions where they approach or traverse bone 41. Regional variations in cell morphology and glycosaminoglycan content in tendons have been reported 42 as well as in the canine ACL suggesting that the ligament is subjected to multiaxial stresses 43. In the present study, we did not discriminate between different regions of native ligament so the higher levels of fibrocartilaginous proteins may arise from the origin and insertion regions of the ACL. Nevertheless sGAG analysis, histological and immunohistochemical staining of aggrecan and versican in the ACL mid‐substance indicates that these proteins are also upregulated throughout the entire ligament. The higher proportion of collagen type III, aggrecan, and versican observed in ligament agrees with a previous comparison between human ACL and patellar tendon 44.

In the current study, 3D TE constructs were created from mature canine LDET and ACL fibroblasts using in vitro 3D cell fibrin cultures 22. Constructs from both tissues displayed a high degree of cellularity and collagen fibril content. Collagen fibrils were mainly located in extracellular space, but were also occasionally found in plasma membrane protrusions also known as fibripositors found previously in embryonic tendon 39, 45. The proteomic comparison between native TL tissue and 3D TE constructs demonstrated significantly more ECM proteins in native tissues, while both 3D tissues had more cellular associated proteins. The higher levels of cellular associated proteins in 3D TE constructs were likely to be due to the greater cell content in these tissues compared to the native tissues. Their high DNA and low collagen content is indicative of a high cell‐to‐matrix ratio. In contrast 3D TE constructs were also found to contain high sGAG content, suggesting that the proteoglycans are rapidly acquired and mature much faster or may require less maturation than the collagen matrix. Proteoglycans have been shown to play role in regulation of tendon collagen fibrillogenesis 46 and prevent later fusion of collagen fibrils 47. This higher proteoglycan content (based on sGAG measurement) might play an important role in organization of the collagen fibrils and development of 3D TE constructs. Therefore our findings reflect the immature state of the constructs in this study and are consistent with previous observations in adult human tendon constructs 28, 48. To date, the extent of maturation of 3D TE T/L constructs is not fully known. However, Herchenhan et al. 48 demonstrated a fivefold and 50% increase of mechanical strength and collagen fibril diameter when constructs were subjected to static tension for 5 weeks. Other studies have identified that factors such as uniaxial cyclic stretch 49 and addition of growth factors such as transforming growth factor 1 or insulin growth factor 1 23, 27 can increase collagen gene expression, content, and fibril diameter in 3D fibrin constructs.

PCA analysis of our proteomic data suggested that there were no statistically significant differences between 3D T/L constructs. This was in contrast to native T/L, which separated into distinct groups, based upon their protein content. These findings suggest that fibroblasts of the T/L do not result in distinct 3D constructs during the 14‐day culture period we have used. This might indicate that cell source is not an important factor for tissue engineering although longer term in vitro studies with more mature constructs would be required to test this. Proteomic composition between 3D T/L constructs that were derived from T/L cell source indicate that tendon and ligament fibroblasts are not phenotypically distinct when cultured in vitro. This data indicate that fine‐tuning ECM composition may be more significant challenge for tendon and ligament tissue engineering. It is yet to be determined whether T/L cells become a tendon/ligament or whether different cell sources such mesenchymal stem cells or skin dermal fibroblasts differentiate into ligament or tendon when situated in their native in vivo environment.

A possible limitation to this study is the chondroitinase ABC treatment step that was included to produce a better protein separation of proteoglycan peptides and to facilitate trypsin digestion. The extreme charge density of the long GAG chains could have interfered with protein separation during ion exchange chromatography and could have reduced the efficiency of trypsin digestion, or introduced unwanted variability at this crucial step. However, the chondroitinase treatment could have also extracted other chondroitinase sulfate binding proteins that may have been overlooked. It should also be considered that while most proteins were solubilized in all samples, a fraction of proteins was insoluble and retained in pellet form. This is most likely due to cross‐linked collagen chains (Peffers et al. 29). Future studies may involve optimization of protein extraction using a combination of other chaotropic agents and LC‐MS/MS analysis on the insoluble fraction.

In conclusion, we have shown for the first time the differences between tendon, ligament, and 3D TE tendon and ligament constructs. Our findings make a vital contribution to future tendon and ligament tissue engineering and regeneration strategies.

*The authors have declared no conflict of interest*.

## Supporting information

As a service to our authors and readers, this journal provides supporting information supplied by the authors. Such materials are peer reviewed and may be re‐organized for online delivery, but are not copy‐edited or typeset. Technical support issues arising from supporting information (other than missing files) should be addressed to the authors.

Supplementary Information
