# Supplementary material for: Proteomic differences between native and tissue‐engineered tendon and ligament
Source: Proteomics. 2016 May 11;16(10):1547–56. doi: 10.1002/pmic.201500459 (PMC5132062; doi:10.1002/pmic.201500459)
Supplement: Supplementary file 1 — Supplementary Information [file PMIC-16-1547-s001.zip › pmic12319-sup-0001-text.docx]

**Tendon and ligament 3D tissue engineered construct formation**

To isolate tendon and ligament cells, LDET and ACL that were sectioned into small pieces of around 1 mm^3^ and digested overnight in DMEM (Life technologies, MD, USA) containing 0.1% collagenase (Worthington, Lakewood, NJ) and 10% FBS (Gibco, Paisley, UK). Cells in the 2^nd^ and 3^rd^ passage were used for the construct set up. Briefly, each well of a six-well plate was coated with ~1.5ml SYLGARD (WPI, Hertfordshire, UK) and pinned with silk sutures to create fixed anchor points. For creation of three technical fibrin constructs, 1.2mls of 1.5x10^6^ cells/ml were suspended into 250µl of 20mg/ml fibrinogen and 25µl of 200U/ml thrombin (both Sigma-Aldrich, Dorset, UK) added. 480µl of the mixture was immediately deposited in each well and vigorously shaken to ensure an even covering of the fibrin gel. Each cell- embedded fibrin gel was cultured in 2 mL phenol-red free DMEM supplemented with 100 units/ml penicillin/streptomycin, 10% FBS (Gibco, Paisley, UK), 500ng/ml amphotericin, 2 mM L-glutamine (both life technologies, Paisley, UK) 200 µM l-ascorbic acid 2-phosphate (Sigma-Aldrich, Dorset, UK), non-essential amino acid (Sigma-Aldrich, Dorset, UK) at 10µl/ml concentration and aprotinin (Sigma-Aldrich, Dorset, UK) at 10 µl/ml. Constructs were incubated at 37°C with 5% CO_2_. Every 48 hours, media was replaced and construct were scored with a fine pipette tip to detach adhesions to the side of the well. All constructs were fully contracted between the anchored points and were harvested at 14 days post-seeding.

**Liquid chromatography and tandem mass spectrometry**

Samples were run in random order on 1 hour gradient with an inter-sample 30 minutes blank to ensure for no sample carryover. To evaluate instrument performance, standards of Ecoli digests spiked with RePLiCal were included at the start and end of the run. Of each sample aliquots (10µl) of tryptic peptides equivalent to 93 ng proteins per sample were loaded onto a trap column (Acclaim PepMap 100, C18, 20 mm x 75 µm) at a flow rate of 5 µL/min with 0.1% (v/v) TFA and 2%(v/v) acetonitrile. After 3 min, the trap column was set in-line with an analytical column (Easy-Spray PepMap® C18, 15 cm x 75 µm, 2 µm). Peptide elution was carried out using a gradient mixture of solvent A (HPLC grade water with 0.1%(v/v) formic acid) and solvent B (HPLC grade acetonitrile 80%(v/v). Separations were performed by applying a linear gradient of 3.8% to 50% solvent B over 30 minutes at 300 nL/min followed by a washing step (5 minutes at 99% solvent B) and an equilibration step (10 minutes at 3.8% solvent B). The Q-Exactive instrument was operated in data dependent positive (ESI+) mode acquiring full MS scan (*m/z* 300-2000) at 70,000 resolution (*m/*z 200) after accumulation of ions to 1x10^6^ target value based on predictive automatic gain control values from the previous full scan. Dynamic exclusion was set to 20s. The 10 most intense multiply charged ions (*z* ≥ 2) were sequentially isolated and fragmented in the octopole collision cell by higher energy collisional dissociation (HCD) with a fixed injection time of 100ms and 35,000 resolution. Typical mass spectrometric conditions were as follows: spray voltage, 1.9kV, no sheath or auxillary gas flow; heated capillary temperature, 250°C; normalised HCD collision energy 30%. The MS/MS ion selection threshold was set to 1 x 10^4^ counts and a 2 *m/z* isolation width was set.

**Immunohistochemistry**

Sequential sections of 4 μm from paraffin-embedded samples were deparaffinised and rehydrated in two washes of xylene, 100% and 95% ethanol solution for 10 minutes and in two washes of ultrapure water for 5 minutes. Endogenous peroxidase activity was blocked by incubating the tissue sections with 3% H_2_O_2_ for 10 minutes. After endogenous peroxidase block, the slides were washed two times for 5 minutes in water and then in tris buffered saline (TBS) for an additional 5 minutes. Sections for asporin, versican and agreccan immunostaining were pre-digested with chondroitinase ABC (Sigma-Aldrich, Dorset, UK) (0.5 U/ml) in 100 mM Tris-HCL pH 7.2-7.4 for 30 minutes at room temperature followed by two washes of 5 minutes in TBS. Each section was subsequently blocked with 200 µl of 10% normal goat serum (PCN500, Invitrogen, CA, USA) for 1 hour at room temperature to prevent non-specific antibody binding. The solution was replaced with 100µl of primary antibody diluted in TBS. The slides were incubated overnight at 4°C. After incubation, the sections were washed for three times for 5 minutes in TBS and incubated with appropriate of secondary antibody for 1 hour at room temperature. For the primary antibodies that were raised in rabbit, 100µl of Zytochem Plus HRP polymer goat anti-rabbit was used as secondary antibody (ZUC032, Zytomed system, DE). For the primary antibodies that were raised in mouse, 100µl goat antimouse was used (A4416, Sigma-Aldrich, UK) as secondary antibody.

Following incubation with secondary antibody, the slides were washed three times in TBS before adding 400µl of 3,3- diaminobenzidine (Sigmafast DAB, Sigma-Aldrich, Dorset, UK) for 1 minute. The slides were immersed in ultrapure water for 5 minutes, counterstained with Mayer's Haemalum for 1 minute and washed in tap water. The sections were then dehydrated and mounted with di-N-Butyle Phthalate in Xylene (D.P.X.) (Sigma-Aldrich, Dorset, UK).

| **Primary antibody** | **Manufacturer** | **Secondary antibody** |
| --- | --- | --- |
| Collagen III (1:100) | Abcam (ab7778) | ZytoChemPlus (HRP) Polymer anti-Rabbit (ZUC032) |
| Asporin (1:100) | Abcam (ab58741) | ZytoChemPlus (HRP) Polymer anti-Rabbit (ZUC032) |
| Agreccan (7D1) (1:50) | Donated by B.Caterson/C. Hughes | Anti-Mouse IgG  (1:50) (A4416, Sigma, UK) |
| Versican (1:100) | Hybridoma (12C5) | Anti-Mouse IgG  (1:50) (A4416, Sigma, UK) |

Primary and secondary antibodies used for immunohistochemistry staining.
