# Supplementary material for: Proteomic differences between native and tissue‐engineered tendon and ligament
Source: Proteomics. 2016 May 11;16(10):1547–56. doi: 10.1002/pmic.201500459 (PMC5132062; doi:10.1002/pmic.201500459)
Supplement: Supplementary file 1 — Supplementary Information [file PMIC-16-1547-s001.zip › pmic12319-sup-0002-text.docx]

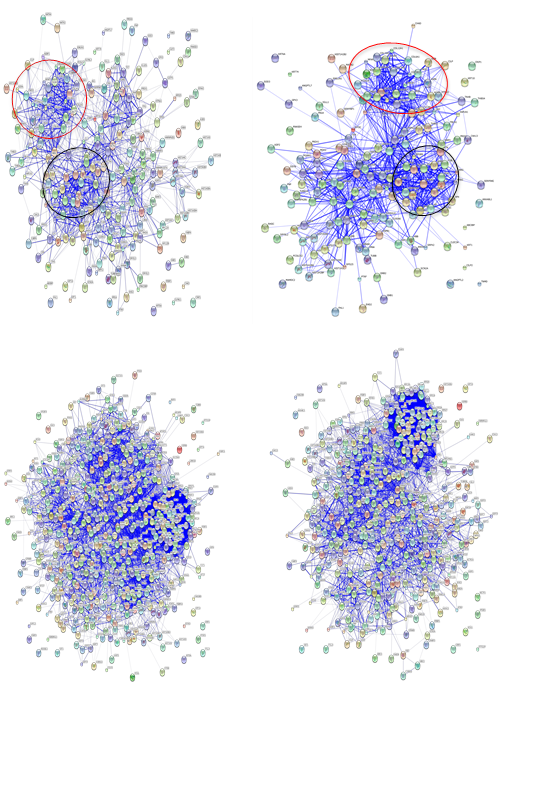


Supplementary Figure 1. String analysis of native tendon (A), native ligament (B), 3D tendon construct (C) and 3D ligament construct (D). The principal gene ontology processes for native tendon and native ligament were identified as ECM organisation (p=2.1e^-28^, p=3.75e^-27^), wound healing (p=8.7e-^15^, p=1.85e-^14^) and collagen fibril organisation (p=1.5e^-13^, p=1.19e^-13^). In both native tendon and ligament ECM proteins (red circle) and matrisome associated proteins (black circle) have the most evident protein-protein interaction. For 3D tendon and ligament constructs the main principal gene ontology processes are translational elongation (p=2.3e^-63^, p=3.71e^-65^) and protein targeting to ER (p=1.75e-^65^, p=9.98e^-64^).
